# Enhanced recovery after surgery reduces postoperative nausea and vomiting in gynecological surgery: systematic review and meta-analysis of randomized and observational studies

**DOI:** 10.3389/fmed.2026.1909915

**Published:** 2026-08-12

**Authors:** Xin Zhou, Mengyu Zhang, Ping Zheng

**Affiliations:** 1 Department of Anesthesiology, West China Hospital, Sichuan University/West China School of Nursing, Sichuan University, Chengdu, Sichuan, China; 2 West China Tianfu Hospital, Sichuan University, Chengdu, Sichuan, China

**Keywords:** enhanced recovery after surgery, gynecological surgery, meta-analysis, multimodal analgesia, perioperative care, postoperative nausea and vomiting, systematic review

## Abstract

**Background:**

Enhanced recovery after surgery (ERAS) protocols have been increasingly adopted in the perioperative management of gynecological conditions. However, the aggregate impact of ERAS on postoperative nausea and vomiting (PONV) following gynecological surgery remains incompletely characterized.

**Objective:**

To evaluate the effectiveness of ERAS protocols in reducing PONV incidence among patients undergoing gynecological surgery.

**Methods:**

This systematic review and meta-analysis was conducted in accordance with the PRISMA 2020 and MOOSE guidelines. A comprehensive literature search was performed across Web of Science, PubMed, Embase, and ScienceDirect from the inception of the databases up to April 2026. The Cochrane Risk of Bias 2 (RoB 2) tool was used for RCTs, and the Newcastle-Ottawa Scale (NOS) was used for cohort studies. The primary outcome was PONV incidence following gynecological surgery. Pooled effect estimates were expressed as odds ratios (ORs) with 95% confidence intervals (CIs). Robustness was assessed through sensitivity analysis. Publication bias was evaluated using funnel plots, Begg’s test, and Egger’s test.

**Results:**

Eighteen studies (11 RCTs and 7 cohort studies), encompassing 3,275 patients and conducted between 2014 and 2026, were included. Fixed-effects meta-analysis demonstrated that ERAS significantly reduced PONV incidence compared with conventional care (OR = 0.48, 95% CI = 0.40 to 0.58, *P* < 0.001). Statistical heterogeneity was low (*I*^2^ = 29.8%, *P* = 0.114). Subgroup analysis revealed that the mean BMI was the possible source of heterogeneity (interaction *P*-value = 0.045). Neither influential studies nor significant publication bias were identified.

**Conclusion:**

Implementation of ERAS protocols significantly reduces PONV following gynecological surgery. Widespread adoption of standardized ERAS pathways is essential to optimize postoperative recovery and alleviate the burden of PONV in this patient population.

**Systematic review registration:**

https://www.crd.york.ac.uk/PROSPERO/view/CRD420261359744, identifier CRD420261359744.

## 1 Introduction

Enhanced recovery after surgery (ERAS) represents a comprehensive, multidisciplinary perioperative care paradigm that integrates evidence-based interventions to optimize the entire surgical trajectory, spanning from preoperative preparation through postoperative rehabilitation (1). These protocols have been implemented as a quality improvement strategy, demonstrating substantial benefits for both patient outcomes and healthcare system efficiency (2). Initially pioneered for colorectal procedures in Europe (3), ERAS protocols have subsequently been extended to a diverse range of surgical disciplines, including orthopedic, urologic, thoracic, and obstetric surgery (4, 5). In gynecology, ERAS has been adopted for the management of both benign and malignant conditions (1). The first ERAS Society guidelines for gynecologic oncological surgery, published in 2016, showed significant agreement with colorectal surgery recommendations (1). However, unlike colorectal interventions, gynecological procedures have unique anatomical features, surgical methods, and complication profiles that require specialized attention (6).

Despite the expanding adoption of ERAS in gynecology, the supporting evidence base remains limited. Postoperative nausea and vomiting (PONV) is a prevalent complication among female patients undergoing gynecological procedures, with the reported incidence ranging from 15 to 43% (7). Opioids are a cornerstone of perioperative analgesia; however, their administration is often accompanied by a range of adverse sequelae, including respiratory depression, nausea, and vomiting (8). Notably, prospective randomized controlled trials specifically evaluating the efficacy of ERAS in gynecologic surgery remain scarce (9), underscoring the need for robust evidence synthesis to validate these protocols in this specific surgical context.

Patients undergoing gynecological surgery are particularly susceptible to PONV, with an estimated incidence approaching 50% (10). This elevated prevalence reflects the convergence of multiple independent risk factors inherent to this population, including female sex, non-smoking status, and postoperative opioid requirements (11). Female sex is recognized as an independent predictor of PONV and serves as a critical determinant in preoperative risk stratification (12). Given the significant impact of PONV on patient comfort, satisfaction, and recovery trajectory, international guidelines advocate for a multimodal preventive strategy incorporating both pharmacological and non-pharmacological interventions (13). ERAS protocols offer a structured, evidence-based approach to PONV mitigation. Low-opioid anesthesia, a pivotal element of multimodal analgesia within ERAS, has emerged as a prominent area of investigation. Evidence indicates that this strategy attenuates PONV, reduces postoperative delirium, and accelerates recovery (14). Furthermore, ERAS guidelines recommend the initiation of a normal diet within the first 24 h post-operatively, positing that adequate nutritional support facilitates the restoration of bowel function and reduces the hospital length of stay (5).

Although individual ERAS components show promise, the overall impact of these integrated pathways on PONV in gynecological surgery remains incompletely defined. Therefore, we conducted this systematic review and meta-analysis to assess the effectiveness of ERAS protocols in reducing the incidence of PONV among patients undergoing gynecological surgery, thus guiding the improvement of perioperative care standards.

## 2 Materials and methods

### 2.1 Study design and reporting guidelines

This systematic review and meta-analysis was conducted in accordance with the Preferred Reporting Items for Systematic Reviews and Meta-Analyses (PRISMA) 2020 statement and Meta-analysis of Observational Studies in Epidemiology (MOOSE) guidelines (15, 16). The study protocol was prospectively registered with PROSPERO (registration number: CRD420261359744; registered date: April 4, 2026).

### 2.2 Eligibility criteria

Studies were eligible for inclusion if they met the following criteria: (1) Population: Adult patients (aged ≥18 years) undergoing elective gynecological surgery, including procedures for both benign and malignant conditions; (2) Intervention: Implementation of a multimodal ERAS protocol. For this review, an ERAS protocol was defined as a structured care pathway incorporating at least three of the following key elements: preoperative counseling and optimization, avoidance of prolonged fasting (e.g., carbohydrate loading), multimodal opioid-sparing analgesia, maintenance of normothermia, goal-directed fluid therapy, early mobilization, or early resumption of oral intake; (3) Comparator: Patients receiving conventional perioperative care; (4) Outcomes: Reported data on the incidence of postoperative nausea and vomiting (PONV); and (5) Study design: Randomized controlled trials (RCTs) and observational cohort studies. All surgical approaches, including open (laparotomy), laparoscopic, and robotic-assisted procedures, were included.

Studies were excluded if they met any of the following criteria: (1) Participants were pediatric patients or underwent emergency surgery; (2) The intervention involved a single isolated perioperative element rather than a multimodal ERAS protocol; (3) They were laboratory-based or preclinical research, reviews, case reports, or conference abstracts; (4) They were non-English language publications; or (5) The full text was unavailable.

### 2.3 Search strategy

The literature search was structured according to the PICO (Population, Intervention, Comparator, Outcome) framework, focusing on patients undergoing gynecological surgery (P), ERAS implementation (I), conventional perioperative care (C), and PONV incidence (O). The following electronic databases were systematically searched from the inception of the databases up to April 2026: Web of Science, PubMed, Embase, and ScienceDirect. Additionally, the reference lists of relevant reviews and meta-analyses were manually screened to identify potentially eligible studies. The search strategy employed the following query: {(Enhanced Recovery After Surgery OR Enhanced Postsurgical Recovery OR ERAS OR enhanced recovery) AND [[(Postoperative Nausea and Vomiting) OR Nausea AND Vomiting] OR PONV OR Postoperative Nausea OR Postoperative Emeses OR Postoperative Emesis OR Postoperative Vomiting] AND (Gynecologic Surgical Procedure OR Gynecological Surgery OR Gynecological Surgeries OR Gynecologic Surgery OR Pelvic Floor Disorders OR Pelvic Organ Prolapse)}. The detailed electronic search strategies for each database are provided in Supplementary Table 1.

### 2.4 Study selection

Following database retrieval, duplicate records were removed using EndNote reference management software (Clarivate Analytics, Philadelphia, PA, USA). Two reviewers independently screened titles and abstracts for relevance to the study objectives. Subsequently, the full texts of potentially eligible studies were assessed against the predefined inclusion and exclusion criteria. Discrepancies between reviewers were resolved through consensus discussion or adjudication by a third reviewer.

### 2.5 Quality assessment

The methodological quality of the 11 included randomized controlled trials (RCTs) was independently evaluated by two reviewers using the Cochrane Risk of Bias 2 (RoB 2) tool (17). The quality of the 7 cohort studies was assessed using the Newcastle-Ottawa Scale (NOS) (18). The NOS consists of 7 items, with most items being scored 1 point; however, the comparability item can receive a maximum of 2 points. Studies scoring below 5 were considered to be at high risk of bias.

### 2.6 Data extraction and synthesis

Data extraction was independently conducted by two reviewers using a standardized data collection form. Discrepancies were resolved through discussion or adjudication by a third reviewer. The following variables were extracted: first author, publication year, country, study design, sample size, mean age, mean body mass index, and surgical procedure. Pooled effect estimates were presented as odds ratios (ORs) with 95% confidence intervals (CIs). Statistical heterogeneity was quantified using the *I*^2^ statistic and Cochran’s Q test. Thresholds of 25, 50, and 75% were interpreted as low, moderate, and high heterogeneity, respectively. A random-effects model was employed when moderate to high heterogeneity was detected; otherwise, a fixed-effects model was applied. Influential studies were identified through sensitivity analysis. Publication bias was assessed using funnel plot visualization, Begg’s test, and Egger’s test.

### 2.7 Statistical analysis

All statistical analyses were conducted using Stata 16.0 (StataCorp, College Station, TX, USA). For dichotomous outcomes, the Mantel–Haenszel (M-H) method was used to pool odds ratios (OR) with 95% confidence intervals (CIs). The primary analysis utilized a fixed-effects model. To assess robustness, a random-effects model was also applied. Studies with zero events were included in the analysis using the Mantel–Haenszel method without arbitrary continuity correction. The “meta,” “metainf,” “metafunnel,” and “metabias” commands were utilized for pooled analyses, sensitivity analyses, and publication bias assessments, respectively. A two-tailed *P*-value < 0.05 was considered statistically significant.

## 3 Results

### 3.1 Study selection

**FIGURE 1:**
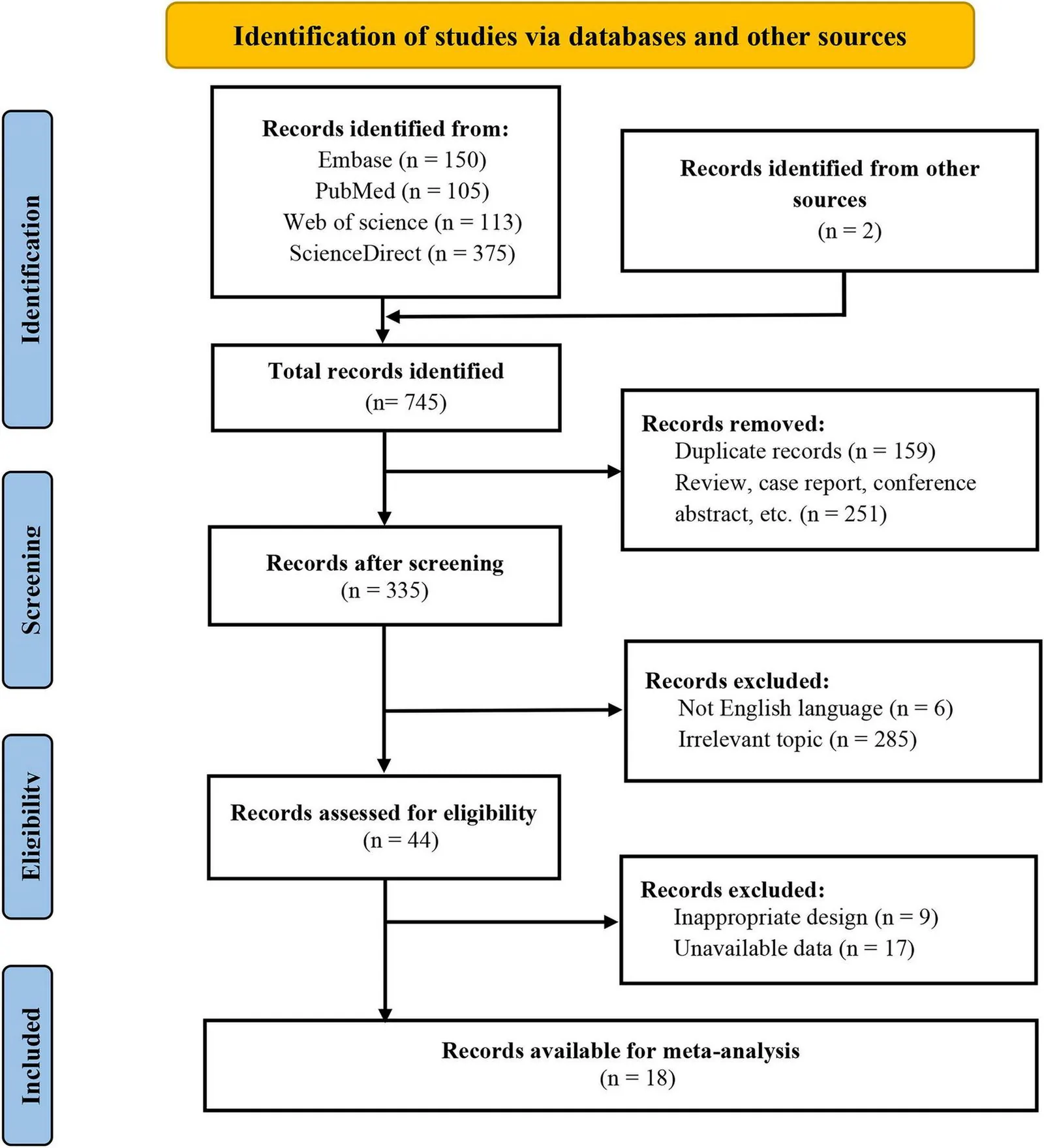
PRISMA flowchart of 18 included studies.

The PRISMA flow diagram of study selection is presented in Figure 1. The initial database search identified 743 potentially relevant citations, and 2 additional studies were identified through manual reference list screening. After title and abstract screening, 44 articles were assessed for full-text eligibility. After full-text review, 18 studies met the inclusion criteria and were incorporated into the final meta-analysis (19–36).

### 3.2 Study characteristics and quality assessment

The 18 included studies consisted of 11 RCTs and 7 cohort studies, which were conducted between 2014 and 2026. In total, these studies involved 3,275 patients. The sample sizes of individual studies ranged from 60 to 439 participants. The mean age of patients ranged from 34 to 68 years, and the mean body mass index ranged from 22 to 34 kg/m^2^. Detailed study characteristics are summarized in Table 1.

**TABLE 1:** Summarized characteristics of 18 included studies.

References	Country	Design	Simple size	Mean age	Mean BMI	Surgery
Bahadur et al. (19)	India	RCT	130	47	27	Hysterectomy
Deng et al. (20)	China	RCT	74	50	24	Sacrocolpopexy
Erkan et al. (21)	Turkey	RCT	100	50	28	Hysterectomy
Ferrari et al. (22)	Italy	RCT	168	55	25	Hysterectomy
Huang et al. (23)	China	RCT	95	68	24	PFR
Lindemann et al. (24)	Norway	Cohort	439	64	25	Laparotomy
Peng et al. (25)	China	RCT	130	47	24	Gynecological oncology surgery
Ren et al. (26)	China	RCT	144	40	23	Gynecologic laparoscopic surgery
Reuter et al. (27)	Germany	Cohort	134	60	25	Gynecological oncology surgery
Sang et al. (28)	China	Cohort	402	42	22	Focused ultrasound ablation surgery
Sulima et al. (29)	Russia	Cohort	60	34	25	Pelvioperitonitis surgery
Suresh Kumar et al. (30)	India	RCT	279	53	27	Gynecological oncology surgery
Teigen et al. (31)	USA	RCT	118	30	34	Cesarean
Wijk et al. (32)	Sweden	Cohort	205	55	26	Hysterectomy
Xiao et al. (33)	China	Cohort	80	61	22	PFR
Xing et al. (34)	China	RCT	340	44	25	Gynecological surgery
Yan et al. (35)	China	RCT	257	67	24	PFR
Yu et al. (36)	China	Cohort	120	42	23	Hysterectomy

PFR, pelvic floor reconstruction surgery.

Methodological quality assessment of the 11 RCTs using the RoB 2 tool revealed that, given the nature of the interventions, blinding of participants and personnel was challenging, and deviations from intended interventions could not be entirely precluded. Consequently, all included RCTs were judged to raise “some concerns” regarding risk of bias (Supplementary Figure 1). The 7 cohort studies all achieved NOS scores of at least 5 stars, and none were excluded based on quality criteria (Supplementary Table 2).

### 3.3 Effectiveness of ERAS in preventing PONV

**FIGURE 2:**
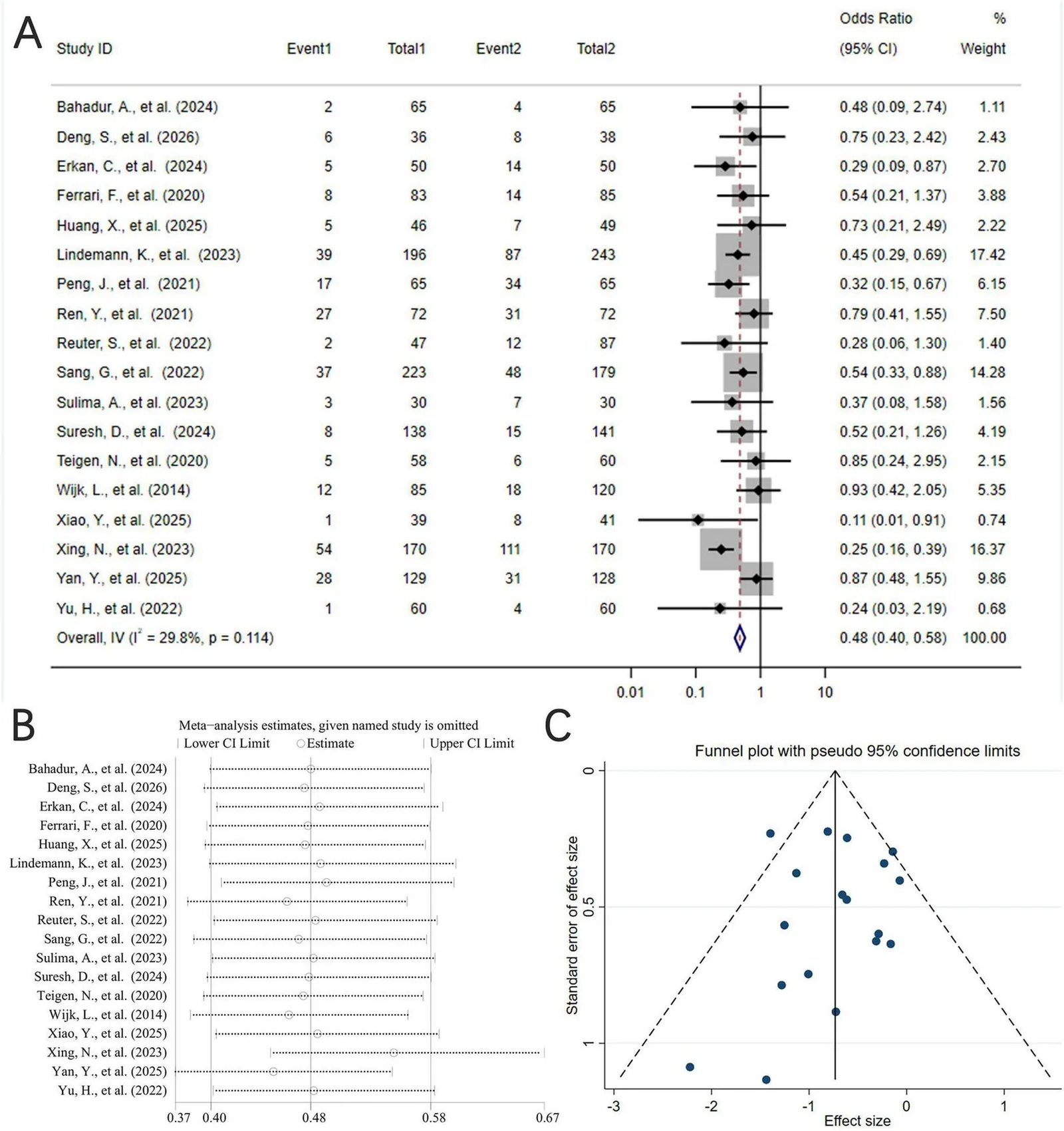
ERAS can effectively prevent PONV after gynecological surgery. **(A)** Forest plot of the risk of PONV after gynecological surgery; **(B)** sensitivity analysis for the risk of PONV; **(C)** funnel plot for assessing publication bias.

Fixed-effects meta-analysis of the 18 included studies (19–36) demonstrated that ERAS significantly reduced the incidence of PONV following gynecological surgery compared with conventional care (OR = 0.48, 95% CI = 0.40 to 0.58, *P* < 0.001). Statistical heterogeneity was low (*I*^2^ = 29.8%, *P* = 0.114) (Figure 2A). Sensitivity analysis identified no influential studies (Figure 2B). Furthermore, no significant publication bias was detected (Figure 2C; Begg’s test: *z* = −0.91, *P* = 1.000; Egger’s test: *t* = 0.03, *P* = 0.979).

To address the robustness of our findings, we also performed a random-effects model analysis. We found that ERAS still reduced the incidence of PONV (OR = 0.49, 95% CI = 0.39 to 0.63, *P* < 0.001). The results of the subgroup analysis revealed that the mean BMI was the possible source of heterogeneity. Patients with a mean BMI ≥ 25 kg/m^2^ (OR = 0.41, 95% CI = 0.32 to 0.52) had a lower OR than those with a mean BMI < 25 kg/m^2^ (OR = 0.59, 95% CI = 0.45 to 0.78, interaction *P*-value = 0.045). However, the mean age (interaction *P*-value = 0.413), country (interaction *P*-value = 0.773), study design (interaction *P*-value = 0.800) and sample size (interaction *P*-value = 0.838) were not possible sources of heterogeneity (Table 2).

**TABLE 2:** Subgroup analyses for sources of heterogeneity.

Potential factors	Subgroup	Effect (95% CI)	*I* ^2^	Interaction *p*-value
Mean age	< 60 years	0.46 (0.37, 0.57)	29.9%	0.413
	> 31 years	0.54 (0.39, 0.74)	37.7%	
Mean BMI	< 25 kg/m^2^	0.59 (0.45, 0.78)	17.4%	**0.045**
	≥ 25 kg/m^2^	0.41 (0.32, 0.52)	23.1%	
Country	Asian countries	0.47 (0.38, 0.59)	47.5%	0.773
	Western countries	0.50 (0.37, 0.68)	0.0%	
Study design	RCT	0.47 (0.37, 0.60)	45.2%	0.800
	Cohort	0.49 (0.37, 0.66)	0.0%	
Sample size	< 100	0.52 (0.26, 1.03)	0.0%	0.838
	≥ 100	0.48 (0.40, 0.58)	38.7%	
Total effect	Fixed-effect	0.48 (0.40, 0.58)	29.8%	–
	Random-effect	0.49 (0.39, 0.63)	29.8%	–

BMI, body mass index; RCT, randomized controlled trial. Bold value indicates statistical significance (*P* < 0.05).

## 4 Discussion

Implementation of ERAS protocols represents a pivotal advancement in perioperative care, aiming to optimize the entire surgical trajectory through a multidisciplinary, evidence-based approach. ERAS protocols commence with preoperative planning and extend through postoperative rehabilitation until the patient achieves full recovery and discharge (37). These comprehensive pathways encompass a wide array of perioperative interventions designed to optimize patient outcomes (Table 3). Preoperative measures include patient education and counseling, prehabilitation, modified bowel preparation, shortened fasting periods with carbohydrate loading, venous thromboembolism prophylaxis, and surgical site infection prevention strategies. The latter includes antimicrobial prophylaxis, skin antisepsis, normothermia maintenance, avoidance of routine drainage, and perioperative glycemic control. Intraoperative and postoperative components encompass selection of appropriate anesthetic techniques and minimally invasive surgical approaches, goal-directed fluid therapy, opioid-sparing multimodal analgesia, early nutritional support, prevention of postoperative ileus and nausea/vomiting, and promotion of early mobilization (19–36).

**TABLE 3:** Comprehensive perioperative interventions in ERAS protocols.

Surgery	Preoperative	Intraoperative	Postoperative	References
Hysterectomy	Optimized Fasting and carbohydrate loading, thromboembolism, avoidance of routine bowel preparation, patient education	Multimodal and opioid-sparing analgesia, normothermia and fluid management, PONV prophylaxis	Early oral intake, early mobilization, early catheter removal	(19, 21, 22, 32, 36)
Laparotomy	Optimized fasting and carbohydrate loading, patient education	Multimodal and opioid-sparing analgesia, maintenance of normothermia, avoidance of abdominal drains	Early oral intake, early mobilization, early catheter removal	(24)
Oncology surgery	Optimized fasting and carbohydrate loading, avoidance of routine bowel preparation, thromboembolism and infection prophylaxis	Goal-directed fluid therapy, maintenance of normothermia, multimodal analgesia	Early oral intake, early mobilization, early device removal, nausea and vomiting control	(25, 27, 30)
Laparoscopic surgery	Optimized fasting and carbohydrate loading, thromboembolism and infection prophylaxis	Normothermia and fluid management, PONV prophylaxis	Early oral intake, early catheter removal, early mobilization	(26)
PFR	Optimized fasting and carbohydrate loading, preemptive analgesia, avoidance of routine bowel preparation, intravenous access and monitoring	Total intravenous anesthesia, intraoperative fluid restriction, multimodal analgesia	Multimodal analgesia, early oral intake, early catheter removal, postvoiding residual test	(20, 23, 33, 35)

PFR, pelvic floor reconstruction surgery.

Implementation of enhanced recovery pathways for patients undergoing minimally invasive gynecologic oncology surgery has been significantly associated with reduced postoperative length of stay, decreased healthcare expenditures, and improved analgesic efficacy (38). Despite widespread adoption of ERAS across surgical disciplines, its implementation in gynecological surgery remains sub-optimal. Surveys assessing ERAS clinical pathways in gynecologic oncology have identified a substantial implementation gap: merely 21% of hospitals reported adoption rates exceeding 80%, with only 8.4% achieving rates above 90% (39). This is particularly concerning given the high symptom burden frequently associated with advanced gynecologic conditions, including nausea and impaired gastrointestinal function, which ERAS pathways are specifically designed to mitigate (40). The primary focus of ERAS in gynecology is the management of PONV, a prevalent complication that significantly impedes recovery and the transition to ambulatory care settings. Excessive opioid utilization is associated with adverse effects including impaired gastrointestinal motility, PONV, respiratory depression, and delayed mobilization, ultimately prolonging the postoperative recovery process (41). Consequently, ERAS protocols emphasize an opioid-sparing strategy through multimodal analgesia, which has been demonstrated to substantially reduce both the incidence and severity of PONV (25). For instance, a randomized controlled trial involving elderly patients undergoing transvaginal pelvic floor reconstruction demonstrated that ERAS implementation significantly reduced postoperative length of stay and pain scores, facilitated opioid-sparing analgesia, and decreased PONV incidence (23). In this context, implementation of multimodal analgesia to minimize opioid consumption and alleviate postoperative pain resulted in a substantial reduction in both PONV incidence and severity (11). Furthermore, PONV mitigation likely reflects the combined effects of accelerated mobilization, prompt enteral feeding, gum chewing, and opioid-sparing strategies implemented within the first 48 h postoperatively (27).

The findings of this meta-analysis provide robust evidence supporting the efficacy of ERAS protocols in reducing PONV. Our analysis of 18 studies demonstrated that ERAS implementation significantly lowers PONV incidence following gynecological surgery compared with standard care (OR = 0.48, 95% CI = 0.40 to 0.58, *P* < 0.001). The low statistical heterogeneity and absence of significant publication bias strengthen the validity and generalizability of this conclusion. Notably, the results of the subgroup analysis revealed that patients with a mean BMI ≥ 25 kg/m^2^ had a lower OR than those with a mean BMI < 25 kg/m^2^ (interaction *P*-value = 0.045). Current literature supports that implementing ERAS pathways effectively mitigates the burden of PONV among obese patients undergoing metabolic procedures (42). These results underscore the success of the multimodal strategy inherent to ERAS, particularly the reduction in opioid consumption, which constitutes a primary driver of postoperative vomiting and intestinal dysfunction (43). Furthermore, adherence to a standardized protocol ensures a more consistent perioperative trajectory and contributes to efficient resource utilization, with no detrimental impact on postoperative rehabilitation (5). Although ERAS protocols yield certain economic advantages, the additional expenditures required for multimodal analgesia may partially offset these benefits, resulting in comparable total hospitalization costs (44).

In conclusion, this meta-analysis provided robust evidence that implementation of ERAS protocols significantly reduced PONV incidence following gynecological surgery. This benefit is largely attributable to the protocol’s emphasis on opioid-sparing multimodal analgesia. The certainty of the evidence was formally assessed using the GRADE methodology, revealing moderate overall quality. Therefore, broader implementation of standardized ERAS pathways is essential to improve postoperative care, reduce patients’ symptom burden, and increase the feasibility of conducting ambulatory gynecologic surgery.

## 5 Limitations

Several limitations should be considered when interpreting the findings of this meta-analysis. First, substantial heterogeneity exists in the documentation and reporting of adverse events across the included ERAS evaluations, which hampers direct comparisons and may affect the consistency of pooled estimates. Variations in outcome definitions, assessment tools, and monitoring protocols likely contributed to this inconsistency. Second, the unblinded design of the included studies represents an unavoidable methodological constraint inherent to the nature of the intervention. Additionally, since most surgical procedures were scheduled in advance, potential temporal confounding cannot be excluded. Third, although we assessed publication bias using funnel plots, Begg’s test, and Egger’s test, the statistical power of these tests is limited when the number of included studies is small. Therefore, the absence of significant publication bias in subgroup analyses with fewer studies should be interpreted with caution. Finally, the generalizability of our findings may be limited by differences in local protocols, patient populations, and healthcare settings across the included studies. Future research should prioritize standardized reporting protocols and employ rigorous study designs to enhance the quality and comparability of evidence.

## 6 Conclusion

ERAS protocols significantly reduce PONV incidence after gynecological surgery. The reduction in PONV may be attributable, at least in part, to opioid-sparing multimodal analgesia incorporated within ERAS pathways. Widespread implementation of standardized ERAS pathways is crucial to optimize postoperative recovery and reduce the symptom burden caused by PONV. Future efforts must prioritize improving protocol adherence to ensure high-quality outcomes for all patients undergoing gynecological procedures.

## Data Availability

The original contributions presented in this study are included in this article/Supplementary material, further inquiries can be directed to the corresponding author.

## Supplementary material

Supplementary Figure 1 Bias risk summary for the 11 included RCTs based on the RoB 2 tools.
