# Supplementary material for: Enhanced recovery after surgery reduces postoperative nausea and vomiting in gynecological surgery: systematic review and meta-analysis of randomized and observational studies
Source: Front Med (Lausanne). 2026 Aug 12;13:1909915. doi: 10.3389/fmed.2026.1909915 (PMC13506897; doi:10.3389/fmed.2026.1909915)
Supplement: Supplementary file 1 [file Data_Sheet_1.docx]

Table S1. Search strategy for the databases.

| Databases | Search strategy | Published date | Results |
| --- | --- | --- | --- |
| PubMed | ((Enhanced Recovery After Surgery OR Enhanced Postsurgical Recovery OR ERAS OR enhanced recovery) AND (Postoperative Nausea and Vomiting OR Nausea and Vomiting OR PONV OR Postoperative Nausea OR Postoperative Emeses OR Postoperative Emesis OR Postoperative Vomiting)) AND (Gynecologic Surgical Procedure OR Gynecological Surgery OR Gynecological Surgeries OR Gynecologic Surgery OR Pelvic Floor Disorders OR Pelvic Organ Prolapse) | 1948-2026 | 105 |
| Web of science | Enhanced Recovery After Surgery OR Enhanced Postsurgical Recovery OR ERAS OR enhanced recovery (Topic) AND Postoperative Nausea and Vomiting OR Nausea and Vomiting OR PONV OR Postoperative Nausea OR Postoperative Emeses OR Postoperative Emesis OR Postoperative Vomiting (Topic) AND Gynecologic Surgical Procedure OR Gynecological Surgery OR Gynecological Surgeries OR Gynecologic Surgery OR Pelvic Floor Disorders OR Pelvic Organ Prolapse (Topic) | 1900-2026 | 113 |
| ScienceDirect | ((“Enhanced Postsurgical Recovery” OR “ERAS”) AND (“Nausea and Vomiting” OR “PONV” OR “Postoperative Emeses”)) AND (“Gynecologic Surgical Procedure” OR “Gynecological Surgery” OR “Pelvic Floor Disorders” OR “Pelvic Organ Prolapse”) | 1997-2026 | 375 |
| EMBASE | ((Enhanced Recovery After Surgery or Enhanced Postsurgical Recovery or ERAS or enhanced recovery) and ((((Postoperative Nausea and Vomiting) or Nausea) and Vomiting) or PONV or Postoperative Nausea or Postoperative Emeses or Postoperative Emesis or Postoperative Vomiting) and (Gynecologic Surgical Procedure or Gynecological Surgery or Gynecological Surgeries or Gynecologic Surgery or Pelvic Floor Disorders or Pelvic Organ Prolapse)).af. | 1974-2026 | 150 |
