# Supplementary material for: Enhanced recovery after surgery reduces postoperative nausea and vomiting in gynecological surgery: systematic review and meta-analysis of randomized and observational studies
Source: Front Med (Lausanne). 2026 Aug 12;13:1909915. doi: 10.3389/fmed.2026.1909915 (PMC13506897; doi:10.3389/fmed.2026.1909915)
Supplement: Supplementary file 2 [file Data_Sheet_2.docx]

| Table S2. Quality assessment based on the Newcastle-Ottawa Scale (NOS) checklist. | | | | | | | | | | |
| --- | --- | --- | --- | --- | --- | --- | --- | --- | --- | --- |
| **Study** | **Selection** | | | |  | **Comparability** |  | **Outcome** | | **Total score** |
|  | Representativeness of the sample | Sample size | Non-respondent | Ascertainment of the exposure |  | Confounding factors are controlled |  | Assessment of outcomes | Statistical test |  |
| Lindemann, K., et al. 2023 | * | * | * | * |  | * |  | * | * | 7 |
| Reuter, S., et al. 2022 | * | * | * | * |  | * |  | * | * | 7 |
| Sang, G., et al. 2022 | * | * | * | * |  | ** |  | * | * | 8 |
| Sulima, A., et al. 2023 | * | * | * | * |  | - |  | * | * | 6 |
| Wijk, L., et al. 2014 | * | * | * | * |  | * |  | * | * | 7 |
| Xiao, Y., et al. 2025 | * | * | * | * |  | ** |  | * | * | 8 |
| Yu, H., et al. 2022 | * | * | * | * |  | - |  | * | * | 6 |
